# *Respice...prospice: *Philosophy, ethics and medical care- past, present, and future.

**DOI:** 10.1186/1747-5341-5-17

**Published:** 2010-11-09

**Authors:** James Giordano

**Affiliations:** 1 Potomac Institute for Policy Studies 901 N. Stuart St. Suite 900 Arlington, VA 22203, USA; 2 Oxford Centre for Neuroethics Uehiro Centre for Practical Philosophy University of Oxford OX1, 1PT Oxford, UK

## *Respice*,* adspice*,* prospice*: (Latin) Look to the past, and the present, in order to gauge the future

In an accompanying interview, Prof. Edmund Pellegrino reflects upon a life in medicine and bioethics, and offers his perspectives on the current and future state of these fields. Professor Pellegrino reiterates his oft-cited view of the inextricability of philosophy, ethics and the humanities in science and clinical medicine. This intertwinement must be acknowledged and regarded in any consideration of the nature and extent of the myriad possibilities and problems that can and will arise in medicine, and in the moral decisions mandated by its circumstances. *Philosophy, Ethics and Humanities in Medicine (PEHM) *responds to these realities by providing a forum for deep discussion of the philosophical bases of science and medicine, so as to attempt to depict 1) the intensity and complexity of the intersection of philosophy and ethics; 2) how this intersection is revealed in research and therapeutics, and 3) how philosophical premises might ground ethical analyses and approaches that are necessary for sound medical care. Simply put, the vision and mission of *PEHM *is to generate thought and reflection upon the richness of the situations and relationships that are intrinsic to medicine.

The notion of philosophy, ethics and humanities *in *medicine is important, as it communicates an appreciation for ethics and humanitarian concern(s) as essential to each and all of the dimensions that contribute to and constitute medicine as a profession and practice. The papers appearing in *PEHM *illustrate this, and also seek to inform, educate, and at times, provoke debate and controversy. This year has seen a fine complement of papers: some re-examining longstanding questions and constructs of medicine [1-3]; others focusing upon issues that reveal the shifting exigencies and contingencies of healthcare in an evermore technololgic and globalized world culture [4-10], and still others that look to philosophy and history to portend the potential constructs, contexts and concerns that will establish the ethical landscape of medicine in years to come [11-14] Certain papers remain the focus of discourse - and dialectic - for some time as the issues they raise seethe anew in the crucible of professional, public and/or political conversation. To be sure, this has been the case with Prof. Thomas Papadimos' essay "Healthcare Access as a Right not a Privilege: A Construct of Western Thought" [15], that has stimulated ongoing deliberation and debate upon putative right to medical care, the relationship of ethics to policy, and the assertion of Aristotelian philosophical claims to healthcare-as-right.

Each and all of these themes are relevant - and controversial - in light of recent healthcare reforms and the changing climate of healthcare provision. There is some question as to whether Aristotle actually endorsed healthcare as a right, and Prof. Pellegrino (working with the staff of the National Reference Center for Bioethics Literature at the Kennedy Institute for Ethics at Georgetown University) has raised this point by noting specific citations from Annas, Long, Miller, Schofield, et al. [16] The reader is recommended to this work (*vide infra*), as both commentary upon the ongoing discussion of the issue at hand - and Prof. Papadimos' paper.

Pellegrino calls for "...broader conversation...about the whether any classical foundation exists for the right to healthcare", and recently, John K. Hall and Mark V. Boswell have asserted that while physicians - and, might I add, patients - may like to think of healthcare as a human right, ethics and law do not provide clear support for this position [17]. As these authors note, there is little to support the provision - and goodness - of medical care beyond the moral premises of the traditional physician-patient relationship. In examining and directing the fiduciary nature and power of this relationship, we may return to Aristotle: for while he may not have explicitly described healthcare as a right, he most certainly advocated moral acumen and responsibility in its act(s) of profession [18].

But it is important to bear in mind that legislators do not render patient care, clinicians do, and I urge that we view this task scopiously, so as to obtain an enhanced appreciation for the professional responsibilities for healthcare that arise from what the most current scientific findings - and the humanities - reveal about health, disease and illness. This may provide deeper insight to why and how the realties - and philosophy and ethics - of medicine should inform healthcare policy. Toward these ends, it might be meaningful to reflect upon the teachings of the past. Aristotle's depiction of the interdependence of science, practical wisdom, philosophy, ethics, and politics is just as relevant today as it was centuries ago [19]. It speaks to the reciprocity between these disciplines and pursuits to inform the public and the professions, and at the same time be informed by - and sensitive to - the public nature of the human predicament when making those decisions that affect and sustain the social value of health and healthcare.

As Aristotle recognized, a healthy citizenry makes for a stronger republic. But achieving such goals is reliant, at least in part, upon responsibilities borne by the "strong" (viz.- those in power, whether medical, economic, and/or political) to mitigate the plight of the weak (viz.- those who are made vulnerable and marginalized by disease, illness and suffering). Yet, whatever the system of medical care may turn out to be, it will be enacted in an increasingly technophilic milieu that is steeped in - and influenced by - a market mindset that is quick to commoditize resources, services and goods. To ethically navigate this socio-economic landscape, clinicians might do well to heed Aristotle's call for virtue and temperance, for despite the fact that technology has dramatically enhanced the quality of health and healthcare, the imprudent use of any technology - old or new- can be problematic, if not (pragmatically and ethically) erroneous [20-22]. The goal therefore, is maximize both the right and morally "good" use(s) of technology. To do so, old(er) or low-tech approaches should not be discarded simply because they are, in fact 'not new', nor should new(er) or high-tech methods be blindly accepted (or rejected) merely because of their novelty [20]. Rather, medicine focused upon the best interests of the patient necessitates consideration of both older and newer techniques and technologies, as appropriate.

This decision remains the province of the clinician, in concert with individual patients' needs and values. Yet, the latitude to make such decisions - and provide such care - must be enabled by guidelines, policy and law. Medicine - as profession and practice - does not occur in a socio-political vacuum, and as David Nash has stated, it is important to understand "...the political, educational and economic forces that are helping shape the future practice of medicine..." [23]. To do so will require insight to the philosophy, ethics and humanity of medicine as well as prudence in translating these premises and precepts into the mechanisms, intricacies and effect(s) of policy. Aristotle well-articulated the wisdom and moral courage required engage these tasks by observing that "... to do [right] to the right person, to the right extent, at the right time, with the right motive, and in the right way, that is not for every one, nor is it easy" [19].

Clinicians, philosophers, ethicists, and policy-makers must be well-informed of the facts, circumstances, needs and values of involved agents, and the contingencies, exigencies and potential consequences that are both intrinsic to medicine, and that could be affected by guidelines and reforms. That such policy reforms have been posed reveals an awareness of changing problems and current inadequacies within the systems and conduct of medicine, and an expanding conventional wisdom that has identified the need for health care to "work better" [24-26]. My hope is that *PEHM *can facilitate such positive change by providing a forum for intellectual reflection and discussion that gives rise to fresh ideas and directions that are required if meaningfully sound progress - if not true paradigmatic shift - are to occur. Thus, in some small way, our work together in this journal might contribute to the preservation of something old and valuable (i.e. - perdurable philosophical, ethical and humanitarian constructs) as we forge ahead and shape a new, viable -and better - medicine for the future.
